# The Effect of Non‐Nutritive Sweeteners' Consumption on Body Weight: A Randomized‐Controlled Trial

**DOI:** 10.1002/fsn3.70691

**Published:** 2025-07-24

**Authors:** Mira Daher, Elham El Darazi, Mohammad Kacim, Maya Hobeika, Yonna Sacre

**Affiliations:** ^1^ Department of Nutritional Sciences, Faculty of Health Sciences University of Balamand Koura Lebanon; ^2^ Department of Nutrition and Food Sciences, Faculty of Arts and Sciences Holy Spirit University of Kaslik Jounieh Lebanon; ^3^ Department of Nutrition, Faculty of Public Health Lebanese University Fanar Lebanon; ^4^ Department of Nutrition and Food Sciences, Faculty of Public Health Université Sainte Famille Batroun Lebanon; ^5^ Department of Mathematics, Faculty of Arts and Sciences Holy Spirit University of Kaslik Jounieh Lebanon; ^6^ Department of Biology, Faculty of Arts and Sciences Holy Spirit University of Kaslik Jounieh Lebanon

**Keywords:** body mass index, body weight, non‐nutritive sweetener, obesity, stevia, sucralose

## Abstract

For several decades, non‐nutritive sweeteners (NNS) have been used as alternatives to sugar for caloric control, but their metabolic effects remain unclear. In the literature, they were blamed to contribute to several conditions, including obesity. While their effect on body weight has been investigated in numerous studies, the results have been inconsistent. Our study aims to investigate the effect of NNS consumption on body weight, and whether the results vary among different types of sweeteners. A randomized‐controlled trial was conducted on a final number of 20 healthy participants over a period of 6 weeks, with an initial screening session and two follow‐up sessions. Anthropometric measurements were taken at each visit. While NNS consumers were randomly assigned to the sucralose (0.507 mg/kg; *n* = 7) or stevia group (0.375 mg/kg; *n* = 6), non‐consumers were allocated to the control group (no sweeteners; *n* = 7); all were given an isocaloric diet and were counseled to follow a healthy lifestyle. Our results showed that weight and body mass index significantly decreased among controls (*p*_weight_ = 0.026; *p*_body mass index_ = 0.02) and sucralose (*p*_weight_ = 0.028; *p*_body mass index_ = 0.017), but not among stevia consumers (*p*_weight_ = 0.183; *p*_body mass index_ = 0.138). No significant difference was found between groups where no group had a benefit over the other in terms of weight loss. Moreover, no favorable effect of one sweetener was reported over the other. In our population, sucralose and stevia consumption contributed to weight loss that reached significance among sucralose consumers, and could therefore be considered safe for weight management when used as a part of a healthy lifestyle.

## Graphical abstract

Non‐nutritive sweeteners have been used as sugar alternatives for caloric control, but their effect on body weight remains controversial. A randomized‐controlled trial was conducted on a final number of 20 healthy Lebanese participants over a period of 6 weeks, with an initial screening session and two follow‐up sessions. Among the population under study, sucralose and stevia were safe for weight management when used as part of a healthy lifestyle.

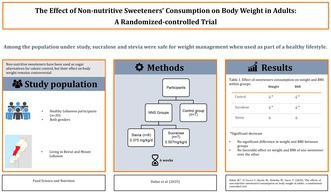

## 1 Introduction

Obesity, a chronic complex disease, has become an epidemic, with the number of individuals affected estimated to exceed one billion worldwide by 2030 (World Health Organization 2023). It is the result of many factors, and causes several medical problems including cardiovascular diseases, cancers, glucose intolerance and type 2 diabetes mellitus (T2D) to cite a few (Nettleton et al. 2016; World Health Organization 2023). Weight management and weight loss are the fundamentals in the treatment of obesity (Cornier 2022) or its related diseases (Shankar et al. 2013), where non‐nutritive sweeteners (NNS) are unintentionally consumed, and have become a popular sugar substitute (Nettleton et al. 2016). Because of their sweet taste and their low caloric contribution, the consumption of that “magical alternative to white sugar” has increased in the past few years; in the United States, the consumption of NNS‐containing beverages has increased from 18.7% to 24.4% among adults, and 33% of women consume food and beverages containing low‐calorie sweeteners (Sylvetsky et al. 2012). In Lebanon, studies addressing the consumption of NNS‐containing items are limited, and show that consumption of such products is prevalent. According to Mousawi et al. (2020), 30% of the population was consuming pills and powders (pp), or products containing NNS. Another study conducted in 2022 revealed that consumption of NNS is highly prevalent among the Lebanese population under study, where 94.4% reported using an item containing NNS at least once in the last 6 months. In the same population, 20.5% were using pp while 94.1% were consuming a NNS‐containing food and beverage (Daher et al. 2022).

Until recently, NNS were thought to be healthy sugar substitutes (Pepino 2015), but it is uncertain whether they can improve metabolic health (Swithers 2013). Their effect on body weight (BW) and obesity has long been investigated, and results were contradictory. While some studies demonstrated that weight loss was equivalent with NNS‐containing beverages and water (Harrold et al. 2023), others have reported an unfavorable effect of NNS (Azad et al. 2017; Bouchard et al. 2010; Pearlman et al. 2017) or NNS‐containing beverages (Fowler et al. 2015; Madjd et al. 2018; Nettleton et al. 2009; Ruanpeng et al. 2017) on BW and obesity. In contrast, some studies found that NNS‐containing beverages do not promote weight gain (Schulze et al. 2004) or had no effect on BW (Bonnet et al. 2018). In addition, other findings reported that NNS consumption decreased BW (Ebbeling et al. 2020; Laviada‐Molina et al. 2020; Rogers and Appleton 2021) and was associated with a lower risk of overweight in women, suggesting it as a potential protective factor against obesity (Duran Aguero et al. 2015).

In Lebanon, studies on the effect of NNS consumption on BW are limited. In addition, human studies specifically assessing the effects of sucralose and stevia are scarce, as most of the existing research focuses on NNS consumption in general without distinguishing between the different types of sweeteners. For this reason, we conducted this study to investigate the effect of NNS consumption, specifically sucralose and stevia, on BW among Lebanese adults residing in Beirut and Mount‐Lebanon, and to investigate if the results vary between different types (natural or artificial) of NNS available in the Lebanese market.

## 2 Materials and Methods

### 2.1 Study Design and Procedure

**FIGURE 1:**
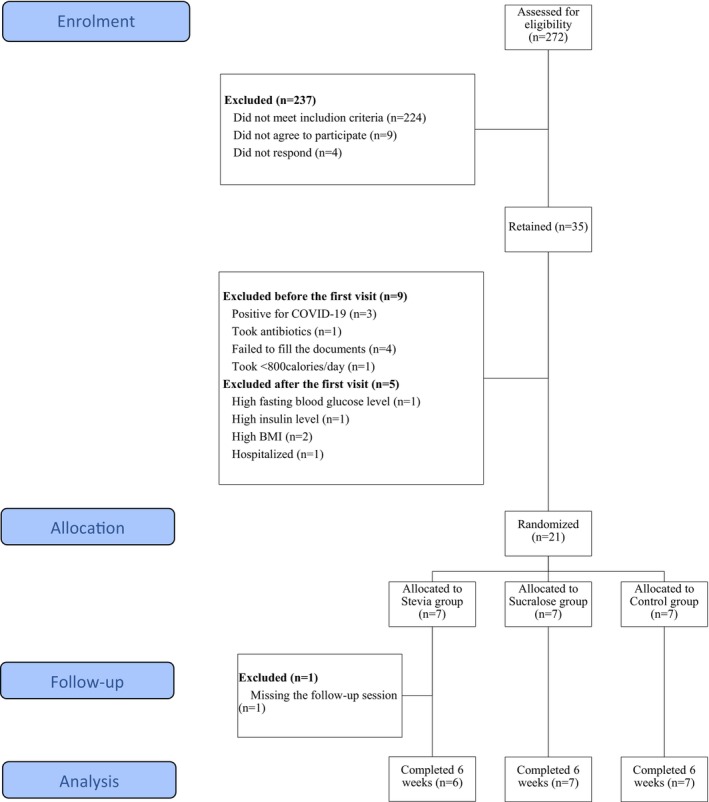
Participant disposition. BMI, body mass index; COVID‐19, coronavirus disease.

The present study was conducted on a final number of 20 healthy Lebanese participants, recruited from Beirut and Mount‐Lebanon, over a period of 6 weeks from May to July 2021. Announcements were posted on social media from January to May 2021, to which 272 participants responded and filled an online screening survey. Participants who met inclusion criteria (Table S1) were contacted and screened to be assigned to one of our three experimental groups; a final number of 35 participants was retained, and individual virtual meetings were conducted, during which the study was explained in detail, individual artificial sweeteners' intake was assessed by filling a validated food frequency questionnaire (FFQ) (Myers et al. 2018), and participants were asked to sign the consent form and to perform a food record over 3 days (two weekdays and one weekend). At the end of this phase, nine subjects were excluded either because they tested positive for coronavirus disease (COVID‐19) (*n* = 3), took antibiotics (*n* = 1), did not fill the requested documents (*n* = 4) or were taking less than 800 cal per day (*n* = 1); 26 participants made it to the first visit. After the first visit, five participants were excluded either because they were found to have a high fasting blood glucose level (*n* = 1), a high insulin level (*n* = 1), a high body mass index (BMI) (*n* = 2), or because they were hospitalized (*n* = 1); 21 participants started the experimental part. At the end of Week 3, one participant was excluded for missing the scheduled follow‐up session. Twenty participants finished the study (Figure 1).

### 2.2 Experimental Protocol

In this study, NNS consumers were randomly assigned to one of the two experimental groups (Group 1: Stevia, Group 2: Sucralose), while non‐consumers were allocated to the control group. All participants underwent a washout period of 2 weeks. A document containing the majority of NNS‐containing products was provided, and all subjects were educated to avoid any product containing NNS except the one prescribed for participants in the experimental groups. An isocaloric diet that matches individual caloric needs was prescribed for each subject.

### 2.3 Procedure

All participants were asked to attend three sessions consisting of one screening session and two follow‐up sessions. During the screening session, anthropometric measurements were taken, and the BMI was calculated. Blood tests and other measurements were conducted to study factors other than obesity, not addressed in this article. A questionnaire was filled out by all participants by a trained dietitian after being tested and verified. NNS consumers were randomly assigned to one of the sweetener groups (*n* = 7 per group) at a dose equivalent to 0.507 mg/kg for sucralose, 0.375 mg/kg for stevia, as obtained from a previous study (Daher et al. 2022). Non‐consumers, defined as individuals consuming less than one food or beverage containing low‐calorie sweeteners per month (Sylvetsky et al. 2017) were assigned to the control group, as many individuals either prefer not to consume sweeteners or dislike their taste, which could affect compliance and consequently the overall results of the study (Peters et al. 2016). Commercially available sucralose and stevia (Steviol glycosides), which were previously found to be commonly used in the population under study (Daher et al. 2022) were used. This allows for a comparison between artificial and natural sweeteners. Participants were instructed about the amount of sweeteners to be consumed per day, and the isocaloric diet was prescribed. They were informed that sweeteners could be taken anytime throughout the day. In addition, they were asked to complete a food record consisting of two weekdays and one weekend and to bring any unused sweetener for follow‐up. Follow‐up sessions were scheduled once every 3 weeks.

### 2.4 Data Tools Collection and Techniques

In this study, the baseline questionnaire covered personal status (age, gender, marital status, educational level and living district), economic status (work and income), daily habits (smoking, alcohol consumption, physical activity levels, and water intake), NNS consumption (current and previous consumption of NNS), medical data, and nutrition data in addition to a 24‐h dietary recall and a FFQ. Moreover, a food record was requested prior to each visit (Fink and Mikesky 2013). During follow‐up visits, a questionnaire covering health and nutrition status was filled out, unused sweeteners were counted, and anthropometrics were taken, in addition to a 24‐h dietary recall.

### 2.5 Dietary Habits

The Mifflin‐St Jeor equation (Mifflin et al. 1990) and the revised Harris‐Benedict equation (Roza and Shizgal 1984) were used to calculate the basal metabolic rate. The mean was calculated, and an isocaloric diet that matches the caloric needs of each participant was prescribed accordingly. The diet was designed following a macronutrient distribution of 50% carbohydrates, 20% proteins, and 30% fats. To make sure that subjects were taking their sweeteners and were following the diet, a 3‐day food record was requested once every 10 days. In addition, unused sweeteners were checked, and a 24‐h dietary recall was conducted at each visit.

### 2.6 Anthropometric Measurements

Anthropometric measurements including weight, height, and BMI were taken. Weight was measured using a medical scale with participants wearing light clothing; they were asked to remove their footwear and stand still (WHO 2005). Measurements were taken in duplicate, and the mean value was considered, to the nearest 0.01 kg. Height was measured using a stadiometer, to the nearest 0.01 cm. Participants were asked to stand and look straight with their feet held together, their heels against the measuring board, and their knees being straight (WHO 2005). The BMI was calculated as the ratio of weight (kg) to the square of height (m). A healthy weight is defined as 18.5 ≤ BMI ≤ 24.9 kg/m^2^, while overweight and obesity are defined as a BMI ≥ 25 kg/m^2^ and ≥ 30 kg/m^2^ respectively (CDC 2021).

### 2.7 Statistical Analysis

The Statistical Package for Social Sciences (SPSS) version 22.0 was used for data entry and analysis. The confidence interval (CI) was set at 95% and significance was considered at *p*‐value < 0.05. Descriptive statistics were performed on data covering personal status (gender, age, marital status, educational level and place of living), economic status (work and income), daily habits (smoking, alcohol consumption, physical activity levels, water intake), BW, and BMI. Results were presented as percentages for qualitative variables and as minimum, maximum, mean, and standard deviation (SD) for quantitative variables. After performing normality and homogeneity tests, paired samples *T*‐test was used to assess the effect of NNS consumption on BW and BMI within groups (within the control group, the sucralose group, the stevia group and all sweeteners group) when the data was normally distributed. When the data was not normally distributed, the non‐parametric Wilcoxon test was used for symmetric data, whereas the bootstrap test was used for non‐symmetric data. Independent samples *T*‐test was used to assess the effect of NNS on those variables between groups (between control and stevia groups, control and sucralose groups, sucralose and stevia groups, as well as between the control and all sweeteners groups) when the data was normally distributed. For non‐normally distributed data, bootstrap was used.

## 3 Results

### 3.1 General Characteristics of the Study Population

Our study included a total of 20 participants (7 males and 13 females), with a mean age of 25.95 ± 4.99 years. Among our participants, 55% (*n* = 11) were living in Mount‐Lebanon, while 45% (*n* = 9) were living in Beirut. The mean value of BW at baseline was 60.59 ± 8.42 kg among controls, 61.74 ± 6.32 kg among the sucralose group, and 62.97 ± 11.51 kg among the stevia group. Participants in the three groups started the study with a normal BMI: controls had a BMI of 22.75 ± 2.18 kg/m^2^, and those in the sucralose and stevia groups had a BMI of 23.27 ± 0.87 and 23.39 ± 2.15 kg/m^2^ respectively. Other characteristics of our population can be found in Table S2.

### 3.2 Effect of NNS Consumption on BW and BMI Within Groups

In this part, we assessed if NNS consumption affected the mean values of BW and BMI within the control group, the sucralose group, the stevia group, in addition to the all sweeteners group, each group apart, over the 6 weeks. It is important to note that the “all sweeteners” group is formed of all participants that consumed either sucralose or stevia over the course of the study (Table 1).

**TABLE 1:** Effect of NNS consumption on body weight and BMI within groups.

	Control			Sucralose			Stevia			All sweeteners		
	Mean ± SD	*N*	p	Mean ± SD	*N*	*p*	Mean ± SD	*N*	*p*	Mean ± SD	*N*	*p*
Weight_W0_ (kg)	60.59 ± 8.42	7	0.096	61.74 ± 6.32	7	0.087	62.97 ± 11.51	6	0.055	62.31 ± 8.70	13	0.014*
Weight_W3_ (kg)	59.77 ± 7.92			60.67 ± 6.86			61.82 ± 11.40			61.20 ± 8.84		
Weight_W3_ (kg)	59.77 ± 7.92	7	0.093	60.67 ± 6.86	7	0.011*	61.82 ± 11.40	6	0.916	61.20 ± 8.84	13	0.194
Weight_W6_ (kg)	58.40 ± 7.26			60.21 ± 6.83			61.73 ± 11.59			60.92 ± 8.94		
Weight_W0_ (kg)	60.59 ± 8.42	7	0.026*	61.74 ± 6.32	7	0.028*	62.97 ± 11.51	6	0.183	62.31 ± 8.70	13	0.02*
Weight_W6_ (kg)	58.40 ± 7.26			60.21 ± 6.83			61.73 ± 11.59			60.92 ± 8.94		
BMI_W0_ (kg/m^2^)	22.75 ± 2.18	7	0.09	23.27 ± 0.87	7	0.049*	23.39 ± 2.15	6	0.039*	23.33 ± 1.52	13	0.009*
BMI_W3_ (kg/m^2^)	22.44 ± 1.95			22.85 ± 0.71			22.94 ± 1.86			22.89 ± 1.30		
BMI_W3_ (kg/m^2^)	22.44 ± 1.95	7	0.013*	22.85 ± 0.71	7	0.01*	22.94 ± 1.86	6	0.772	22.89 ± 1.30	13	0.165
BMI_W6_ (kg/m^2^)	21.93 ± 1.77			22.67 ± 0.76			22.89 ± 1.81			22.77 ± 1.29		
BMI_W0_ (kg/m^2^)	22.75 ± 2.18	7	0.02*	23.27 ± 0.87	7	0.017*	23.39 ± 2.15	6	0.138	23.33 ± 1.52	13	0.010*
BMI_W6_ (kg/m^2^)	21.93 ± 1.77			22.67 ± 0.76			22.89 ± 1.81			22.77 ± 1.29		

Abbreviations: BMI, body mass index; *N*, sample size; SD, standard deviation; W, week.

* Significant difference.

Data analysis indicates that weight loss occurred among all three groups, where the mean value of BW and that of BMI decreased after 6 weeks of intervention. The decrease in BW was found to be significant among controls and the sucralose group at the end of Week 6, where the mean value of BW significantly decreased from 60.59 ± 8.42 to 58.40 ± 7.26 kg (*p* = 0.026), and from 61.74 ± 6.32 to 60.21 ± 6.83 kg (*p* = 0.028) among the controls and sucralose groups respectively. Among the stevia group, values failed to reach significance (*p* = 0.183).

The same was reported for BMI that started to decrease significantly after 3 weeks of intervention, especially among sucralose consumers (*p* = 0.049). When it comes to the stevia group, BMI significantly decreased from 23.39 ± 2.15 kg/m^2^ at baseline to 22.94 ± 1.86 kg/m^2^ (*p* = 0.039) at Week 3.

Surprisingly, the significant decrease in the mean value of BMI among the stevia group was not recorded for BW in the first 3 weeks, but it was near significance where *p* = 0.055. Despite the fact that the mean values of BW and BMI decreased at the end of Week 6 from 62.97 ± 11.51 kg (BMI 23.39 ± 2.15 kg/m^2^) to 61.73 ± 11.59 kg (BMI 22.89 ± 1.81 kg/m^2^), values failed to reach significance among stevia consumers. When we combined stevia and sucralose consumers in one group, the “all sweeteners” group, we found a significant decrease in the mean value of BW and BMI where the mean value of BW significantly decreased from 62.31 ± 8.70 to 60.92 ± 8.94 kg (*p* = 0.02) and that of BMI significantly decreased from 23.33 ± 1.52 to 22.77 ± 1.29 kg/m^2^ (*p* = 0.010).

Those results highlight the fact that weight loss was significant among controls, sucralose, and the all sweeteners group, but not among the stevia group.

### 3.3 Effect of NNS Consumption on BW and BMI Between Groups

In this part, we investigated if there is a significant difference in the mean value of BW and BMI between different groups; we compared the effect of NNS consumption on the mean value of BW and BMI between the control and stevia groups, between the control and sucralose groups, between the sucralose and stevia groups, and between the control and all sweeteners groups at Week 0 (baseline), Week 3, and Week 6.

#### 3.3.1 Difference in BW and BMI Between Control and Stevia Groups

Our results showed that there is a non‐significant difference in the mean value of BW between the control and stevia groups at Week 0 (*p* = 0.670), Week 3 (*p* = 0.717), and Week 6 (*p* = 0.540). The same was reported for the mean value of BMI that did not differ significantly between the controls and stevia consumers at Week 0 (*p* = 0.604), Week 3 (*p* = 0.648), and Week 6 (*p* = 0.356) (Table 2).

**TABLE 2:** Difference in body weight and BMI at Weeks 0, 3, and 6 between control and stevia groups.

Indicator	Groups	*N*	Mean ± SD	*p*
Weight_W0_ (kg)	Control	7	60.59 ± 8.42	0.670
	Stevia	6	62.97 ± 11.51	
Weight_W3_ (kg)	Control	7	59.77 ± 7.92	0.717
	Stevia	6	61.82 ± 11.40	
Weight_W6_ (kg)	Control	7	58.40 ± 7.26	0.540
	Stevia	6	61.73 ± 11.59	
BMI_W0_ (kg/m^2^)	Control	7	22.75 ± 2.18	0.604
	Stevia	6	23.39 ± 2.15	
BMI_W3_ (kg/m^2^)	Control	7	22.44 ± 1.95	0.648
	Stevia	6	22.94 ± 1.86	
BMI_W6_ (kg/m^2^)	Control	7	21.93 ± 1.77	0.356
	Stevia	6	22.89 ± 1.81	

Abbreviations: BMI, body mass index; *N*, sample size; SD, standard deviation; W, week.

#### 3.3.2 Difference in Weight and BMI Between Control and Sucralose Groups

The same was reported while comparing the mean value of BW between the control and sucralose consumers at Week 0 (*p* = 0.798), Week 3 (*p* = 0.821), and Week 6 (*p* = 0.650), and that of BMI which did not differ significantly between the control and sucralose consumers at Week 0 (*p* = 0.569), Week 3 (*p* = 0.618), and Week 6 (*p* = 0.336) (Table 3).

**TABLE 3:** Difference in body weight and BMI at Weeks 0, 3, and 6 between control and sucralose groups.

Indicator	Groups	*N*	Mean ± SD	*p*
Weight_W0_ (kg)	Control	7	60.59 ± 8.42	0.798
	Sucralose	7	61.74 ± 6.32	
Weight_W3_ (kg)	Control	7	59.77 ± 7.92	0.821
	Sucralose	7	60.67 ± 6.86	
Weight_W6_ (kg)	Control	7	58.40 ± 7.26	0.650
	Sucralose	7	60.21 ± 6.83	
BMI_W0_ (kg/m^2^)	Control	7	22.75 ± 2.18	0.569
	Sucralose	7	23.27 ± 0.87	
BMI_W3_ (kg/m^2^)	Control	7	22.44 ± 1.95	0.618
	Sucralose	7	22.85 ± 0.71	
BMI_W6_ (kg/m^2^)	Control	7	21.93 ± 1.77	0.336
	Sucralose	7	22.67 ± 0.76	

Abbreviations: BMI, body mass index; *N*, sample size; SD, standard deviation; W, week.

#### 3.3.3 Difference in BW and BMI Between Sucralose and Stevia Groups

After comparing the difference between control and sweetener groups, we tried to assess if there is a significant difference between different types of sweeteners where sucralose is considered an artificial sweetener while stevia is considered a natural one. Data analysis shows that there is a non‐significant difference in the mean value of BW between sucralose and stevia consumers at Week 0 (*p* = 0.823), Week 3 (*p* = 0.840), and Week 6 (*p* = 0.774). The same was reported for the mean value of BMI that did not differ significantly between the sucralose and stevia consumers at Week 0 (*p* = 0.897), Week 3 (*p* = 0.906), and Week 6 (*p* = 0.792) (Table 4).

**TABLE 4:** Difference in body weight and BMI at Weeks 0, 3, and 6 between sucralose and stevia groups.

Indicator	Groups	*N*	Mean ± SD	*p*
Weight_W0_ (kg)	Sucralose	7	61.74 ± 6.32	0.823
	Stevia	6	62.97 ± 11.51	
Weight_W3_ (kg)	Sucralose	7	60.67 ± 6.86	0.840
	Stevia	6	61.82 ± 11.40	
Weight_W6_ (kg)	Sucralose	7	60.21 ± 6.83	0.774
	Stevia	6	61.73 ± 11.59	
BMI_W0_ (kg/m^2^)	Sucralose	7	23.27 ± 0.87	0.897
	Stevia	6	23.39 ± 2.15	
BMI_W3_ (kg/m^2^)	Sucralose	7	22.85 ± 0.71	0.906
	Stevia	6	22.94 ± 1.86	
BMI_W6_ (kg/m^2^)	Sucralose	7	22.67 ± 0.76	0.792
	Stevia	6	22.89 ± 1.81	

Abbreviations: BMI, body mass index; *N*, sample size; SD, standard deviation; W, week.

#### 3.3.4 Difference in BW and BMI Between Control and All Sweeteners Groups

At the end, we investigated if there is a significant difference in the mean values of BW and BMI between control and all sweeteners groups (sucralose and stevia combined). Data analysis shows that there is a non‐significant difference in the mean values of BW between the control and all sweeteners groups at Week 0 (*p* = 0.672), Week 3 (*p* = 0.723) and Week 6 (*p* = 0.482). The same was reported for the mean value of BMI that did not differ significantly between the control and sweeteners groups at Week 0 (*p* = 0.514), Week 3 (*p* = 0.595) and Week 6 (*p* = 0.274) (Table 5).

**TABLE 5:** Difference in body weight and BMI at Weeks 0, 3, and 6 between control and all sweeteners groups.

Indicator	Groups	*N*	Mean ± SD	*p*
Weight_W0_ (kg)	Control	7	60.59 ± 8.42	0.672
	Sweeteners	13	62.31 ± 8.70	
Weight_W3_ (kg)	Control	7	59.77 ± 7.92	0.723
	Sweeteners	13	61.20 ± 8.84	
Weight_W6_ (kg)	Control	7	58.40 ± 7.26	0.482
	Sweeteners	13	60.92 ± 8.94	
BMI_W0_ (kg/m^2^)	Control	7	22.75 ± 2.18	0.514
	Sweeteners	13	23.33 ± 1.52	
BMI_W3_ (kg/m^2^)	Control	7	22.44 ± 1.95	0.595
	Sweeteners	13	22.89 ± 1.30	
BMI_W6_ (kg/m^2^)	Control	7	21.93 ± 1.77	0.274
	Sweeteners	13	22.77 ± 1.29	

Abbreviations: BMI, body mass index; *N*, sample size; SD, standard deviation; W, week.

## 4 Discussion

NNS have long been used for caloric control but the literature investigating their effect on BW is inconsistent. To assess the effect of NNS consumption on weight, we investigated if BW and BMI changed significantly over 6 weeks of intervention among the control, sucralose, stevia, and all sweeteners groups. Moreover, in order to investigate if a group has an advantage over the other, we compared those parameters between the “control and stevia,” “control and sucralose,” “sucralose and stevia,” and “control and all sweeteners” groups.

A significant weight loss occurred, especially among the control and the sucralose groups, where their BW and BMI significantly decreased after 6 weeks. In the stevia group, values decreased after 6 weeks, without reaching significance. When we combined the stevia and the sucralose groups under the “all sweeteners” group, the mean value of BW and BMI decreased significantly, where NNS consumers lost 1.39 kg after 6 weeks (*p* = 0.02), and their BMI decreased significantly (from 23.33 ± 1.52 to 22.77 ± 1.29 kg/m^2^; *p* = 0.010). Those results show that controls, sucralose, and all sweeteners consumers lost weight except for stevia. Moreover, when we performed a comparison between groups, none of the parameters differed significantly between control and sweeteners. Therefore, NNS consumption did not prevent weight loss nor cause weight gain as claimed in observational studies in particular, and did not have any beneficial nor detrimental effect on BW over controls. In the literature, several authors agree that the beneficial or non‐detrimental effect of NNS on BW was shown in systematic reviews drawing conclusions from clinical trials or intervention studies (Clifton and Fayet‐Moore 2016; Laviada‐Molina et al. 2020; Miller and Perez 2014; Rogers and Appleton 2021), whereas the detrimental effect of NNS on BW was reported in observational studies (Lee et al. 2021; Normand et al. 2021). From our extensive literature review, we support this conclusion, especially that observational studies are known for having the weakest level of evidence where causality cannot be determined, and reverse causality cannot be ruled out (World Health Organization et al. 2022). As for NNS and BW, reverse causality is possible where the weight gain attributed to NNS consumption can be reported to the fact that overweight or obese people tend to consume NNS for caloric control. Therefore, the best level of evidence comes from randomized controlled trials and meta‐analyses performed on those trials. Such studies have reported beneficial (Miller and Perez 2014; Peters et al. 2016; Tate et al. 2012) or neutral effects (Higgins and Mattes 2019; Laviada‐Molina et al. 2020; Bueno‐Hernández et al. 2020; Thomson et al. 2019) on BW which is consistent with our results where a significant weight loss was reported among controls, sucralose, and all sweeteners consumers but not among stevia after 6 weeks. The weight loss reported in our study among controls, sucralose, and all sweeteners consumers was also reported in Tate et al. (2012), where all groups including controls lost a significant weight after 3 and 6 months. Consistently, Miller and Perez (2014) found that NNS consumption decreases BW and BMI without specifying the type of sweeteners studied. Analyzing the effect of NNS consumption on BW by type of sweetener was challenging because the majority of the studies were conducted on low‐calorie sweetened beverages without specifying the type of sweetener used (Miller and Perez 2014), and systematic reviews and meta‐analyses investigating the effect of sucralose or stevia on BW are rare, with the majority conducted on NNS in general or on low‐calorie sweetened beverages.

From the rare studies that investigated the effect of sucralose in particular on weight and BMI, results varied between neutral to no effect; those showed that the decrease in BW among sucralose consumers after 12 weeks was insignificant (Higgins and Mattes 2019), and that BW of sucralose and control groups remained stable across the study (Bueno‐Hernández et al. 2020; Thomson et al. 2019).

When it comes to stevia, only the mean value of BMI decreased significantly in the first 3 weeks without any significant change in BW. After 6 weeks, no significant changes in BW and BMI were reported despite that all values were tending towards decrease, which was consistent with the literature (Higgins and Mattes 2019; Maki et al. 2008). However, the small sample size in the stevia group might have affected the results because when we combined the sweeteners under “all sweeteners” group, the *p*‐value that compared the mean value of BW at baseline to that at Week 6 tended towards more significance and exerted a mild decrease from 0.028 in the sucralose group to 0.020 in the “all sweeteners” group. The same was reported for BMI, where the *p*‐value decreased from 0.017 to 0.010.

In our study, NNS in general and sucralose in particular did not prevent weight loss nor caused weight gain. The reported weight loss can be attributed to the behavioral changes where all participants were asked to follow a healthy lifestyle, and all participants including controls lost weight, which proves that NNS can still lead to weight loss if integrated as part of a behavioral change program. Our results are consistent with the findings of Laviada‐Molina et al. (2020) who found no evidence that NNS consumption promotes weight gain, and with Duran Aguero et al. (2015) who reported that sucralose consumption was associated with a lower risk of overweight in women, making it a protective factor against obesity. However, our findings contradict studies that have linked NNS consumption to obesity through various mechanisms, including overestimation of caloric savings, compensation (Fowler et al. 2008), disruption of energy balance (Peters et al. 2016), making NNS, in our study, a non‐culprit in BW increase.

Interestingly, NNS consumption did not have any favorable effect on BW and BMI over controls; when we compared BW and BMI between control and “stevia,” “sucralose,” and “all sweeteners,” the mean value of those parameters did not differ significantly neither between groups nor between the types of sweeteners. This was consistent with several studies which concluded that there are no significant differences in BW between NNS consumers compared to placebo (Harrold et al. 2023; Maersk et al. 2012; Tate et al. 2012; Toews et al. 2019). Conversely, Stamataki et al. (2020) had different results where controls had a significant increase in BW and BMI when compared to stevia consumers after 6 weeks. However, in their study, no isocaloric diet was given and participants were not required to change their usual diet, which might have affected the results. Moreover, our results removed any role of the type of sweetener in weight loss, consistent with the findings of Higgins and Mattes (2019) at Week 6. However, in their study, sucralose consumers had a significantly lower BW than stevia consumers after 12 weeks, which puts the long‐term difference in the effect of those sweeteners under question.

Our results as a whole indicate that NNS consumption can be an effective method for weight management, but has no advantage over avoiding consumption. The opposite is true, where those who decide to avoid NNS consumption will not have better results than those who use them in their daily life. Moreover, no beneficial effect of one type of sweetener over the other was recorded in our study where BW and BMI did not differ significantly between sucralose and stevia consumers.

The choice of sucralose and stevia among sweeteners was not due to chance. In fact, aspartame in the form of pills or powder is no longer widely available in the Lebanese market, and acesulfame‐K is usually used in combination with other sweeteners in food products (FDA 2025). Therefore, sucralose and stevia were chosen for being commonly used, and because this allows for a comparison between natural and artificial sweeteners. Due to the lack of published data in Lebanon and due to the scarcity of human studies, our study was among the few human studies that investigated the effect of sucralose and stevia separately on BW at a dose representative of the daily dose consumed by individuals living in the area under study. It was the first to compare the different types of sweeteners and the first to be conducted in Lebanon.

However, this study had its limitations, one of which is the sample size. This was addressed by using the appropriate statistical tests and respecting all the assumptions needed to conduct the respective tests, which gives strength to our results. Moreover, compliance of participants made the study harder, but follow‐ups were scheduled regularly to ensure that all participants are following the protocol. In addition, our results could not be extrapolated over the Lebanese population because our sample was selected from Beirut and Mount‐Lebanon. At the end, studies that analyze changes in body composition and that perform gender‐based analysis are needed. Those should be performed over a longer duration and over a larger sample size to investigate the long‐term effect of sucralose and stevia on BW and BMI.

## 5 Conclusion

In conclusion, our study revealed a statistically significant reduction in weight and BMI among participants in the control and sucralose groups, but not in the stevia group. Although the trend within the stevia group was towards decrease, the lack of significance may be attributed to the small sample size within this group because when we combined the sucralose and stevia in one group (all sweeteners group), the *p*‐value approached greater significance. Moreover, no statistically significant differences were observed between the three groups, indicating that neither sucralose nor stevia offered a superior benefit; no group had a benefit over the other in terms of weight loss and BMI decrease, and no favorable effect of one sweetener was reported over the other. Our findings suggest that both sucralose and stevia can be considered to be safe for weight management when used as a part of a healthy lifestyle among the population under study. However, given the small sample size and short duration of this study, further research with a larger sample size is needed to confirm these results and to explore the long‐term effects of the sweeteners on BW and BMI.

This article aligns with Sustainable Development Goal 3: Good Health and Well‐being, as it aims to assess the effect of sweeteners on body weight, especially as obesity is becoming an epidemic across the globe. By understanding one of the multiple factors affecting body weight, our study contributes to global initiatives to decrease the burden of non‐communicable diseases.

## Author Contributions

**Mira Daher:** conceptualization (equal), data curation (equal), formal analysis (equal), funding acquisition (equal), investigation (equal), methodology (equal), project administration (equal), resources (equal), software (equal), validation (equal), visualization (equal), writing – original draft (equal), writing – review and editing (equal). **Elham El Darazi:** writing – review and editing (equal). **Mohammad Kacim:** data curation (equal), formal analysis (equal), writing – original draft (equal), writing – review and editing (equal). **Maya Hobeika:** conceptualization (equal), funding acquisition (equal), methodology (equal), resources (equal), supervision (equal), validation (equal), writing – review and editing (equal). **Yonna Sacre:** conceptualization (equal), funding acquisition (equal), methodology (equal), project administration (equal), resources (equal), supervision (equal), validation (equal), visualization (equal), writing – review and editing (equal).

## Ethics Statement

The study was conducted in accordance with the declaration of Helsinki, and approved by the Institutional Review Board of the Centre Hospitalier Universitaire‐Notre Dame des Secours (reference number 4/2019, on December 20, 2019).

## Consent

Written informed consent was obtained from all study participants. Those gave their consent via the statement “I have read the foregoing information and I consent voluntarily to participate as a participant in this research” where an affirmative reply was required to enter the study. They were able to withdraw from the study at any time without giving a reason. The sweeteners under study were safe for consumption.

## Conflicts of Interest

The authors declare no conflicts of interest.

## Supporting information


Data S1.

## Data Availability

The original contributions presented in this study are included in the article and Supporting Information. Further inquiries can be directed to the corresponding author.

## References

[fsn370691-bib-0002] Azad, M. B. , A. M. Abou‐Setta , B. F. Chauhan , et al. 2017. “Nonnutritive Sweeteners and Cardiometabolic Health: A Systematic Review and Meta‐Analysis of Randomized Controlled Trials and Prospective Cohort Studies.” Canadian Medical Association Journal 189, no. 28: E929–E939. 10.1503/cmaj.161390. 28716847 PMC5515645

[fsn370691-bib-0003] Bonnet, F. , A. Tavenard , M. Esvan , et al. 2018. “Consumption of a Carbonated Beverage With High‐Intensity Sweeteners Has No Effect on Insulin Sensitivity and Secretion in Nondiabetic Adults.” Journal of Nutrition 148, no. 8: 1293–1299. 10.1093/jn/nxy100. 29982723

[fsn370691-bib-0004] Bouchard, D. R. , R. Ross , and I. Janssen . 2010. “Coffee, Tea and Their Additives: Association With BMI and Waist Circumference.” Obesity Facts 3, no. 6: 345–352. 10.1159/000322915. 21196787 PMC6515851

[fsn370691-bib-0005] Bueno‐Hernández, N. , M. Esquivel‐Velázquez , R. Alcántara‐Suárez , et al. 2020. “Chronic Sucralose Consumption Induces Elevation of Serum Insulin in Young Healthy Adults: A Randomized, Double Blind, Controlled Trial.” Nutrition Journal 19, no. 1: 32. 10.1186/s12937-020-00549-5. 32284053 PMC7155288

[fsn370691-bib-0006] CDC . 2021. Defining Adult Overweight and Obesity. Centers for Disease Control and Prevention. https://www.cdc.gov/obesity/adult/defining.html.

[fsn370691-bib-0007] Clifton, P. , and F. Fayet‐Moore . 2016. “Systematic Review of the Safety of Non‐Nutritive Sweeteners.” 10.1016/j.jnim.2015.12.305.

[fsn370691-bib-0008] Cornier, M. 2022. “A Review of Current Guidelines for the Treatment of Obesity.” American Journal of Managed Care 28, no. 15S: S288–S296. 10.37765/ajmc.2022.89292. 36525676

[fsn370691-bib-0009] Daher, M. , C. Fahd , A. A. Nour , and Y. Sacre . 2022. “Trends and Amounts of Consumption of Low‐Calorie Sweeteners: A Cross‐Sectional Study.” Clinical Nutrition ESPEN 48: 427–433. 10.1016/j.clnesp.2022.01.006. 35331524

[fsn370691-bib-0010] Duran Aguero, S. , M. D. Rodríguez Noel , K. Cordón Arrivillaga , et al. 2015. “Association Between Non‐Nutritive Sweeteners and Obesity Risk Among University Students in Latin America [Asociacion entre edulcorantes no nutritivos y riesgo de obesidad en estudiantes universitarios de Latinoamerica].” Revista medica de Chile 143, no. 3: 367–373. 10.4067/S0034-98872015000300012. 26005824

[fsn370691-bib-0011] Ebbeling, C. B. , H. A. Feldman , S. K. Steltz , N. L. Quinn , L. M. Robinson , and D. S. Ludwig . 2020. “Effects of Sugar‐Sweetened, Artificially Sweetened, and Unsweetened Beverages on Cardiometabolic Risk Factors, Body Composition, and Sweet Taste Preference: A Randomized Controlled Trial.” Journal of the American Heart Association 9, no. 15: e015668. 10.1161/JAHA.119.015668. 32696704 PMC7792240

[fsn370691-bib-0012] FDA . 2025. Aspartame and Other Sweeteners in Food. FDA. https://www.fda.gov/food/food‐additives‐petitions/aspartame‐and‐other‐sweeteners‐food.

[fsn370691-bib-0013] Fink, H. H. , and A. E. Mikesky . 2013. Practical Applications in Sports Nutrition. 4th ed. Jones & Bartlett Learning.

[fsn370691-bib-0014] Fowler, S. P. , K. Williams , R. G. Resendez , K. J. Hunt , H. P. Hazuda , and M. P. Stern . 2008. “Fueling the Obesity Epidemic? Artificially Sweetened Beverage Use and Long‐Term Weight Gain.” Obesity 16, no. 8: 1894–1900. 10.1038/oby.2008.284. 18535548

[fsn370691-bib-0015] Fowler, S. P. G. , K. Williams , and H. P. Hazuda . 2015. “Diet Soda Intake is Associated With Long‐Term Increases in Waist Circumference in a Bi‐Ethnic Cohort of Older Adults: The San Antonio Longitudinal Study of Aging.” Journal of the American Geriatrics Society 63, no. 4: 708–715. 10.1111/jgs.13376. 25780952 PMC4498394

[fsn370691-bib-0016] Harrold, J. A. , S. Hill , C. Radu , et al. 2023. “Effects of Non‐Nutritive Sweetened Beverages Versus Water After a 12‐Week Weight‐Loss Program: A Randomized Controlled Trial.” Obesity 31, no. 8: 1996–2008. 10.1002/oby.23796. 37475684

[fsn370691-bib-0017] Higgins, K. A. , and R. D. Mattes . 2019. “A Randomized Controlled Trial Contrasting the Effects of 4 Low‐Calorie Sweeteners and Sucrose on Body Weight in Adults With Overweight or Obesity.” American Journal of Clinical Nutrition 109, no. 5: 1288–1301. 10.1093/ajcn/nqy381. 30997499

[fsn370691-bib-0018] Laviada‐Molina, H. , F. Molina‐Segui , G. Pérez‐Gaxiola , et al. 2020. “Effects of Nonnutritive Sweeteners on Body Weight and BMI in Diverse Clinical Contexts: Systematic Review and Meta‐Analysis.” Obesity Reviews 21, no. 7: e13020. 10.1111/obr.13020. 32216045

[fsn370691-bib-0019] Lee, H. Y. , M. Jack , T. Poon , et al. 2021. “Effects of Unsweetened Preloads and Preloads Sweetened With Caloric or Low‐/No‐Calorie Sweeteners on Subsequent Energy Intakes: A Systematic Review and Meta‐Analysis of Controlled Human Intervention Studies.” Advances in Nutrition 12, no. 4: 1481–1499. 10.1093/advances/nmaa157. 33439973 PMC8321874

[fsn370691-bib-0020] Madjd, A. , M. A. Taylor , A. Delavari , R. Malekzadeh , I. A. Macdonald , and H. R. Farshchi . 2018. “Effects of Replacing Diet Beverages With Water on Weight Loss and Weight Maintenance: 18‐Month Follow‐Up, Randomized Clinical Trial.” International Journal of Obesity 42, no. 4: 835–840. 10.1038/ijo.2017.306. 29633983

[fsn370691-bib-0021] Maersk, M. , A. Belza , H. Stodkilde‐Jorgensen , et al. 2012. “Sucrose‐Sweetened Beverages Increase Fat Storage in the Liver, Muscle, and Visceral Fat Depot: A 6‐mo Randomized Intervention Study.” American Journal of Clinical Nutrition 95, no. 2: 283–289. 10.3945/ajcn.111.022533. 22205311

[fsn370691-bib-0022] Maki, K. C. , L. L. Curry , M. C. Carakostas , et al. 2008. “The Hemodynamic Effects of Rebaudioside A in Healthy Adults With Normal and Low‐Normal Blood Pressure.” Food and Chemical Toxicology 46, no. 7S: S40–S46. 10.1016/j.fct.2008.04.040. 18555574

[fsn370691-bib-0023] Mifflin, M. D. , S. T. St Jeor , L. A. Hill , B. J. Scott , S. A. Daugherty , and Y. O. Koh . 1990. “A New Predictive Equation for Resting Energy Expenditure in Healthy Individuals.” American Journal of Clinical Nutrition 51, no. 2: 241–247. 10.1093/ajcn/51.2.241. 2305711

[fsn370691-bib-0024] Miller, P. E. , and V. Perez . 2014. “Low‐Calorie Sweeteners and Body Weight and Composition: A Meta‐Analysis of Randomized Controlled Trials and Prospective Cohort Studies.” American Journal of Clinical Nutrition 100, no. 3: 765–777. 10.3945/ajcn.113.082826. 24944060 PMC4135487

[fsn370691-bib-0025] Mousawi, M. A. , S. Tlais , A. Alkatib , and H. S. H. Hussein . 2020. “Consumption and Effect of Artificial Sweeteners and Artificially Sweetened Products on Lebanese Population.” International Journal of Environment, Agriculture and Biotechnology 5, no. 4: 882–889. https://journal‐repository.com/index.php/ijeab/article/view/2267.

[fsn370691-bib-0026] Myers, E. A. , E. M. Passaro , and V. E. Hedrick . 2018. “The Comparative Reproducibility and Validity of a Non‐Nutritive Sweetener Food Frequency Questionnaire.” Nutrients 10, no. 3: 334. 10.3390/nu10030334. 29534454 PMC5872752

[fsn370691-bib-0027] Nettleton, J. A. , P. L. Lutsey , Y. Wang , J. A. Lima , E. D. Michos , and D. R. Jacobs Jr. 2009. “Diet Soda Intake and Risk of Incident Metabolic Syndrome and Type 2 Diabetes in the Multi‐Ethnic Study of Atherosclerosis (MESA).” Diabetes Care 32, no. 4: 688–694. 10.2337/dc08-1799. 19151203 PMC2660468

[fsn370691-bib-0028] Nettleton, J. E. , R. A. Reimer , and J. Shearer . 2016. “Reshaping the Gut Microbiota: Impact of Low Calorie Sweeteners and the Link to Insulin Resistance?” Physiology & Behavior 164, no. 4: 488–493. 27090230 10.1016/j.physbeh.2016.04.029

[fsn370691-bib-0029] Normand, M. , C. Ritz , D. Mela , and A. Raben . 2021. “Low‐Energy Sweeteners and Body Weight: A Citation Network Analysis.” BMJ Nutrition, Prevention & Health 4, no. 1: 319–332. 10.1136/bmjnph-2020-000210. PMC8258071 34308140

[fsn370691-bib-0030] Pearlman, M. , J. Obert , and L. Casey . 2017. “The Association Between Artificial Sweeteners and Obesity.” Current Gastroenterology Reports 19, no. 12: 64. 10.1007/s11894-017-0602-9. 29159583

[fsn370691-bib-0031] Pepino, M. Y. 2015. “Metabolic Effects of Non‐Nutritive Sweeteners.” Physiology & Behavior 152: 450–455. 10.1016/j.physbeh.2015.06.024. 26095119 PMC4661066

[fsn370691-bib-0032] Peters, J. C. , J. Beck , M. Cardel , et al. 2016. “The Effects of Water and Non‐Nutritive Sweetened Beverages on Weight Loss and Weight Maintenance: A Randomized Clinical Trial.” Obesity 24, no. 2: 297–304. 10.1002/oby.21327. 26708700 PMC4744961

[fsn370691-bib-0033] Rogers, P. J. , and K. M. Appleton . 2021. “The Effects of Low‐Calorie Sweeteners on Energy Intake and Body Weight: A Systematic Review and Meta‐Analyses of Sustained Intervention Studies.” International Journal of Obesity 45, no. 3: 464–478. 10.1038/s41366-020-00704-2. 33168917

[fsn370691-bib-0034] Roza, A. M. , and H. M. Shizgal . 1984. “The Harris Benedict Equation Reevaluated: Resting Energy Requirements and the Body Cell Mass.” American Journal of Clinical Nutrition 40, no. 1: 168–182. 10.1093/ajcn/40.1.168. 6741850

[fsn370691-bib-0035] Ruanpeng, D. , C. Thongprayoon , W. Cheungpasitporn , and T. Harindhanavudhi . 2017. “Sugar and Artificially Sweetened Beverages Linked to Obesity: A Systematic Review and Meta‐Analysis.” QJM: Monthly Journal of the Association of Physicians 110, no. 8: 513–520. 10.1093/qjmed/hcx068. 28402535

[fsn370691-bib-0036] Schulze, M. B. , J. E. Manson , D. S. Ludwig , et al. 2004. “Sugar‐Sweetened Beverages, Weight Gain, and Incidence of Type 2 Diabetes in Young and Middle‐Aged Women.” Journal of the American Medical Association 292, no. 8: 927–934. 10.1001/jama.292.8.927. 15328324

[fsn370691-bib-0037] Shankar, P. , S. Ahuja , and K. Sriram . 2013. “Non‐Nutritive Sweeteners: Review and Update.” Nutrition 29, no. 11–12: 1293–1299. 10.1016/j.nut.2013.03.024. 23845273

[fsn370691-bib-0038] Stamataki, N. S. , B. Crooks , A. Ahmed , and J. T. McLaughlin . 2020. “Effects of the Daily Consumption of Stevia on Glucose Homeostasis, Body Weight, and Energy Intake: A Randomised Open‐Label 12‐Week Trial in Healthy Adults.” Nutrients 12, no. 10: 3049. 10.3390/nu12103049. 33036155 PMC7600789

[fsn370691-bib-0039] Swithers, S. E. 2013. “Artificial Sweeteners Produce the Counterintuitive Effect of Inducing Metabolic Derangements.” Trends in Endocrinology and Metabolism 24, no. 9: 431–441. 10.1016/j.tem.2013.05.005. 23850261 PMC3772345

[fsn370691-bib-0040] Sylvetsky, A. C. , P. J. Walter , H. M. Garraffo , K. Robien , and K. I. Rother . 2017. “Widespread Sucralose Exposure in a Randomized Clinical Trial in Healthy Young Adults.” American Journal of Clinical Nutrition 105, no. 4: 820–823. 10.3945/ajcn.116.144402. 28228424 PMC5366047

[fsn370691-bib-0041] Sylvetsky, A. C. , J. A. Welsh , R. J. Brown , and M. B. Vos . 2012. “Low‐Calorie Sweetener Consumption is Increasing in the United States.” American Journal of Clinical Nutrition 96, no. 3: 640–646. 10.3945/ajcn.112.034751. 22854409 PMC3417218

[fsn370691-bib-0042] Tate, D. F. , G. Turner‐McGrievy , E. Lyons , et al. 2012. “Replacing Caloric Beverages With Water or Diet Beverages for Weight Loss in Adults: Main Results of the Choose Healthy Options Consciously Everyday (CHOICE) Randomized Clinical Trial.” American Journal of Clinical Nutrition 95, no. 3: 555–563. 10.3945/ajcn.111.026278. 22301929 PMC3632875

[fsn370691-bib-0043] Thomson, P. , R. Santibañez , C. Aguirre , J. E. Galgani , and D. Garrido . 2019. “Short‐Term Impact of Sucralose Consumption on the Metabolic Response and Gut Microbiome of Healthy Adults.” British Journal of Nutrition 122, no. 8: 856–862. 10.1017/S0007114519001570. 31258108

[fsn370691-bib-0044] Toews, I. , S. Lohner , D. Küllenberg de Gaudry , H. Sommer , and J. J. Meerpohl . 2019. “Association Between Intake of Non‐Sugar Sweeteners and Health Outcomes: Systematic Review and Meta‐Analyses of Randomised and Non‐Randomised Controlled Trials and Observational Studies.” British Medical Journal 364: k4718. 10.1136/bmj.k4718. 30602577 PMC6313893

[fsn370691-bib-0045] WHO . 2005. WHO STEPS Surveillance Manual: The WHO STEPwise Approach to Chronic Disease Risk Factor Surveillance/Noncommunicable Diseases and Mental Health, World Health Organization. World Health Organization. http://www.who.int/iris/handle/10665/43376.

[fsn370691-bib-0046] World Health Organization . 2023. “WHO Acceleration Plan to Stop Obesity.” http://www.who.int/iris/handle/10665/370281.

[fsn370691-bib-0047] World Health Organization , M. Rios‐Leyvraz , and J. Montez . 2022. Health Effects of the Use of Non‐Sugar Sweeteners: A Systematic Review and Meta‐Analysis. World Health Organization.

