# Supplementary material for: The Effect of Non‐Nutritive Sweeteners' Consumption on Body Weight: A Randomized‐Controlled Trial
Source: Food Sci Nutr. 2025 Jul 24;13(7):e70691. doi: 10.1002/fsn3.70691 (PMC12288618; doi:10.1002/fsn3.70691)
Supplement: Supplementary file 1 — Data S1. [file FSN3-13-e70691-s001.docx]

Supplementary materials

The present study is part of an overall study assessing the effect of non-nutritive sweeteners consumption on body weight, type 2 diabetes risk factors and gut microbiota composition which explains the reason behind the below inclusion and exclusion criteria.

**Table S.1** Inclusion and exclusion criteria.

| Inclusion criteria | Exclusion criteria |
| --- | --- |
| Age between 18-40 years | Age below 18 or above 40 years |
| Lebanese subjects | Non-Lebanese subjects |
| Subjects who never tested positive for COVID-19 | Subjects who tested positive for COVID-19 |
| Healthy Subjects | Subjects with a major disease or those who underwent a major surgery^†^ |
| Non-diabetic subjects and not taking medications for diabetes | Subjects with diabetes, or taking medications for diabetes |
| Not having risk factors for diabetes | Having risk factors for diabetes |
| Been weight stable of 5kg for the last six months | Subjects who have lost or gained more than 5kg in the last six months |
| Subjects with normal bowel movements (once every 2 days and not more than 3 times per day) | Subjects suffering from constipation or chronic or acute diarrhea within the past four weeks |
| Non-pregnant or lactating women, and not planning to become pregnant in the next three months | Pregnant or lactating women or planning to become pregnant in the next three months |
| Subjects not taking medications | Subjects on hormonal therapy or taking medications^‡^ |

^†^Subjects with a prior diagnosis of cancer, subjects with known gastrointestinal diseases including inflammatory bowel diseases (crohn or ulcerative colitis) or irritable bowel syndrome, subjects with thyroid problems, subjects who were hospitalized or have used systemic antibiotics for more than four weeks, and subjects with a history of bariatric surgery or cholecystectomy; ^‡^Antibiotics (in the last three months), medications that affect weight, metabolism, glucose levels (glucocorticoids, estrogen, androgen), carbohydrates metabolism (thiazide diuretics, glucocorticoids and beta blockers) or immune system, metformin, proton pump inhibitors, H_2_ receptor antagonists, tricyclic antidepressants, narcotics, anticholinergic medications, laxatives, antidiarrheal medications (within four weeks of enrollment), non-steroidal anti-inflammatory drugs, dietary supplements, or antacids (within two weeks of enrollment), and hormonal contraceptives.

**Table S2.** General characteristics of the study population.

| **Variables** | | **n (%)**^†^ |
| --- | --- | --- |
| Sexe | Males | 7 (35%) |
|  | Females | 13 (65%) |
|  | Total | 20 (100%) |
| Age (years) | | 25.95±4.99 |
| Living district | Beirut | 9 (45%) |
|  | Mount-Lebanon | 11 (55%) |
|  | Total | 20 (100%) |
| Baseline body weight (kg) | Controls | 60.59±8.42 |
|  | Sucralose group | 61.74±6.32 |
|  | Stevia group | 62.97±11.51 |
| Baseline BMI (kg/m^2^) | Controls | 22.75±2.18 |
|  | Sucralose group | 23.27±0.87 |
|  | Stevia group | 23.39±2.15 |
| Marital status | Single | 19 (95%) |
|  | Married | 1 (5%) |
|  | Total | 20 (100%) |
| Educational level | Secondary school or below | 2 (10%) |
|  | Bachelor degree | 13 (65%) |
|  | Master’s degree or higher | 5 (25%) |
|  | Total | 20 (100%) |
| Work | Yes | 16 (80%) |
|  | No | 4 (20%) |
|  | Total | 20 (100) |
| Income | ≤675.000L.L^‡^  675.001- 1.499.000L.L^§^ | 1 (6.25%)  5 (31.25%) |
|  | ≥1.500.000L.L^¶^ | 10 (62.5%) |
|  | Total | 16 (100%) |
| Smoking | Currently, daily | 1 (5%) |
|  | Currently, less than daily | 7 (35%) |
|  | Currently, not at all | 12 (60%) |
|  | Total | 20 (100%) |
| Alcohol consumption | **Frequency**  Never  Monthly or less  2-4 times per month  2-3 times per week  Total | 3 (15%)  8 (40%)  7 (35%)  2 (10%)  20 (100%) |
|  | **Amount each time**  0-2 drinks  3 or 4 drinks  Total | 12 (70.6%)  5 (29.4%)  17 (100%) |
| Physical activity | No | 7 (35%) |
|  | Moderately^⁰^ | 13 (65%) |
|  | Total | 20 (100%) |
| Water consumption | <0.5L | 1 (5%) |
|  | 0.5-1L | 5 (25%) |
|  | 1.1-1.5L | 9 (45%) |
|  | >1.5L | 5 (25%) |
|  | Total | 20 (100%) |

Values represent number of respondents (percentage); ^†^Quantitative variables are expressed as mean±SD; ^‡^Equivalent to <448$ before the devaluation of the Lebanese pound; ^§^Equivalent to 448-994$ before the devaluation of the Lebanese pound; ^¶^Equivalent to ≥995$ before the devaluation of the Lebanese pound; ⁰<150minutes/week of moderate-intensity aerobic physical activity or <75minutes of vigorous intensity aerobic physical activity; abbreviations: BMI: body mass index.
